# The evaluation of choroidal vascular index in the patients undergoing surgical treatment for retinal detachment

**DOI:** 10.1007/s10103-026-04816-5

**Published:** 2026-02-04

**Authors:** Dilek Uzlu, İbrahim Mert Kurt, Hidayet Erdöl, Murat Günay, Büşra Köse, Mehmet Kola

**Affiliations:** Department of Ophthalmology, Medical Faculty, Karadeniz Technical University, Trabzon, Turkey

**Keywords:** Intraocular tamponade, Pars plana vitrectomy, Retinal detachment, Subfoveal choroidal vascular index, Visual acuity

## Abstract

To determine the postoperative subfoveal choroidal vascular index (SFCVI) in patients who underwent pars plana vitrectomy (PPV) for rhegmatogenous retinal detachment. Patients who underwent PPV for rhegmatogenous retinal detachment were included in the study. Spectral-domain optical coherence tomography (SD-OCT), including the foveal center, was performed in all patients at the first, third, and sixth months after surgery. This study included 28 patients who underwent PPV for RRD. In 16 eyes, 1000cSt silicone oil (57.1%) and in 12 eyes (42.9%), perfluoropropane (C_3_F_8_) gas were utilized as tamponade agents. A statistically significant difference was observed in the SFCVI value between the C_3_F_8_ and silicone group at the 3rd month (*p* = 0.034), but this difference was not significant in the 6th month (*p* = 0.205). SFCVI was significantly lower in the gas-treated eyes than in the control eyes of the same patients only in the first month (*p* = 0.003), whereas no significant differences were found in other periods. A comparison of the SFCVI values at the first, third, and sixth months in the silicone group with those in the control group revealed no significant differences (*p* = 0.65, 0.52, and 0.50, respectively). There was no significant correlation between SFCVI values and BCVA, IOP, and SFCT values in the postoperative examinations of eyes that underwent PPV due to retinal detachment (*p* > 0.05). Although SFCVI decreases in the early period in patients given C_3_F_8_ as tamponade, it returns to normal levels in the late period. New studies are needed to evaluate the possible functional and anatomical consequences of this temporary decrease in the long term.

## Introduction

 Rhegmatogenous retinal detachment (RRD) is a major cause of visual impairment. It is characterized by the migration of vitreous fluid through a retinal tear into the subretinal space between the neural retina and retinal pigment epithelium (RPE). RRD is a relatively common disease in the general population, occurring in approximately 1/10,000 individuals per year [1]. It is important to restore the anatomical integrity of the macula in a brief period of time in order to ensure the function of the macular region. Pars plana vitrectomy (PPV), scleral buckle, pneumatic retinopexy, or combinations of these methods are used to treat RRD. Various tamponades are preferred for filling the vitreous cavity after PPV. The most frequently used substances are silicone oil and gas (e.g., C_3_F_8_ and SF_6_) [2].

It has been documented that even in the macula with an anatomically normal postoperative appearance, the function of the macula is lower than that of the normal eye, as evidenced by approximately five years of follow-up [3]. A hypothesis has been proposed that this phenomenon is attributable to microstructural alterations in the macula. The aforementioned alterations have been demonstrated to be associated with ischemia and hypoxia. These pathologies result from the disruption of blood flow to the choroid and retina [4, 5]. The choroid is a highly vascularized tissue that nourishes the RPE and outer retina. A decrease in blood flow to the choroid can adversely affect the outer retina, disrupt tissue homeostasis, and cause chorioretinal tissue damage. Many studies have investigated retinal and choroidal thickness changes following retinal detachment surgery [6–10]. The existing literature on this subject is inconclusive, with some studies reporting a decrease in retinal and choroidal thickness after surgery, while others report no change. Furthermore, some studies have demonstrated a decrease in retinal blood flow, as indicated by OCTA [11, 12].

The choroidal vascular index (CVI) is a quantitative assessment technique that provides a quantitative measurement of choroidal vessels by determining the ratio of the choroidal vascular lumen area to the total choroidal area. It is determined based on optical coherence tomography (OCT) images [13]. The CVI was developed as a more sensitive and robust measure of choroidal vascularity than choroidal vessel diameter and choroidal thickness measurements [14]. In comparison with choroidal thickness, CVI has been documented as being less affected by axial length, refractive error, intraocular pressure, and daily biological rhythms [14].

This study aimed to calculate the postoperative subfoveal choroidal vascular index (SFCVI) in patients who underwent PPV for retinal detachment and to investigate the relationship between SFCVI and tamponade type, visual acuity, and subfoveal choroidal thickness. Moreover, the objective was to ascertain whether alterations in SFCVI during postoperative evaluations of patients possess prognostic significance for visual acuity.

## Materials and methods

This retrospective study included patients who underwent PPV for retinal detachment between May 2022 and May 2024 at the Retina Unit of the Department of Ophthalmology, Faculty of Medicine, Karadeniz Technical University. The study was approved by the Karadeniz Technical University Clinical Research Ethics Committee and was conducted in accordance with the Declaration of Helsinki. The patients were informed of the study procedures and provided informed consent.

The medical records of patients who underwent 23-gauge pars plana vitrectomy (PPV) for retinal detachment were retrospectively reviewed. The inclusion criteria were as follows: patients aged > 18 years who had been diagnosed with retinal detachment, underwent surgical treatment, had complete anatomical recovery after treatment, had a follow-up period of at least six months after treatment, and had silicone oil removed before 80 days. Patients with a medical history of previous vitrectomy, preoperative additional posterior segment pathology, and retinal detachment other than RRD (proliferative vitreoretinopathy, traumatic retinal detachment, recurrent cases, cases secondary to macular hole, and detachment associated with high myopia) were excluded from the study. Patients with recurrent retinal detachment, glaucoma, cystoid macular edema, or subretinal fluid during follow-up examinations were excluded from the study. Furthermore, patients who did not comply with the control examination times, those who could not provide adequate cooperation during ophthalmologic examinations, and those with poor image quality on optical coherence tomography (OCT) were also excluded from the study. Among the 112 patients who underwent 23-gauge PPV with a diagnosis of RRD during the study period, 28 patients who met the inclusion criteria were included in the study.

In the present study, 56 eyes from 28 patients were evaluated. The eyes were divided into case and control groups. In case group, the patients were divided into two groups based on the tamponade used: silicone oil (SO) (1000 cSt) or C_3_F_8_ gas and the healthy fellow eyes were evaluated as the control group.

All surgeries were performed under general anesthesia using a standardized 23-gauge three-port pars plana vitrectomy (PPV) system (Alcon Constellation, Alcon, Fort Worth, TX, USA). After core vitrectomy and posterior hyaloid separation was identified using diluted triamcinolone and separated with a vitrector, peripheral vitreous clearance was completed with scleral indentation. The identified tears were marked with diathermy. In all cases, perfluorocarbon liquid was subsequently employed to stabilize the detached retina. The retina was then completely reassured using fluid-air exchange. None of the cases involved peeling of the internal limiting membrane. 360-degree endolaser photocoagulation was applied to the peripheral retina, including the tears. After the perfluorocarbon fluid was completely removed from the vitreous cavity, 1000cSt silicone or C_3_F_8_ (12–15%) was used as a tamponade, depending on the number and location of the tears. Gas tamponade was chosen for breaks above the 8–4 clock-hour meridian, whereas SO was preferred for inferior or multiple tears, giant retinal tears, and in non-compliant patients who could not fully adapt to the head position. The sclerotomies were closed with 8/0 absorbable polyglactin sutures. Patients were advised to maintain the specified head position for at least 10–14 days, depending on the location of the retinal break. Silicone oil removal was accomplished using 23 G three-port access before 80 days postoperatively.

The following data were recorded at the initial and first, third, and sixth months after surgery: age, sex, systemic or ocular diseases, best corrected visual acuity (BCVA) according to Snellen’s chart, intraocular pressure (IOP) (Nidek NT-530, Japan), anteroposterior segment examinations with slit lamp, biometry parameters, and wide-angle fundus photographs (Optos, California, USA). The best-corrected visual acuity obtained using Snellen’s chart was converted to LogMAR for statistical analysis. Subsequent to the surgical procedure, SD-OCT (Solix/Optovue USA) was conducted, encompassing the foveal center, at the 1st, 3rd, and 6th months post-surgery. To ensure consistency and minimize diurnal variability, all OCT scans were performed before noon under standardized lighting conditions by a single technician prior to pupil dilation. Subfoveal choroidal thickness (the distance from the retinal pigment epithelium [RPE] to the chorioscleral junction) was measured manually on the SD-OCT image using the device’s software. All choroidal parameter calculations were performed by the same investigator (İ.M.K.)., who was blinded to the patients’ characteristics. Only scans with the highest quality score (≥ 7 ) were selected for analysis. In this study, data from the silicone, gas, and control groups were compared. The measurements from the silicone and gas groups were also compared with those of the fellow eyes of the patients. The relationship between the time to silicone removal and SFCVI was evaluated. In the silicone group, as in the gas group, measurements were made at 1, 3, and 6 months, but the SFCT and SFCVI values ​​measured at 1 month were not considered because they were made under silicone. Measurements taken after the removal of silicone oil in patients were used in the statistical analysis.

**Fig. 1:**
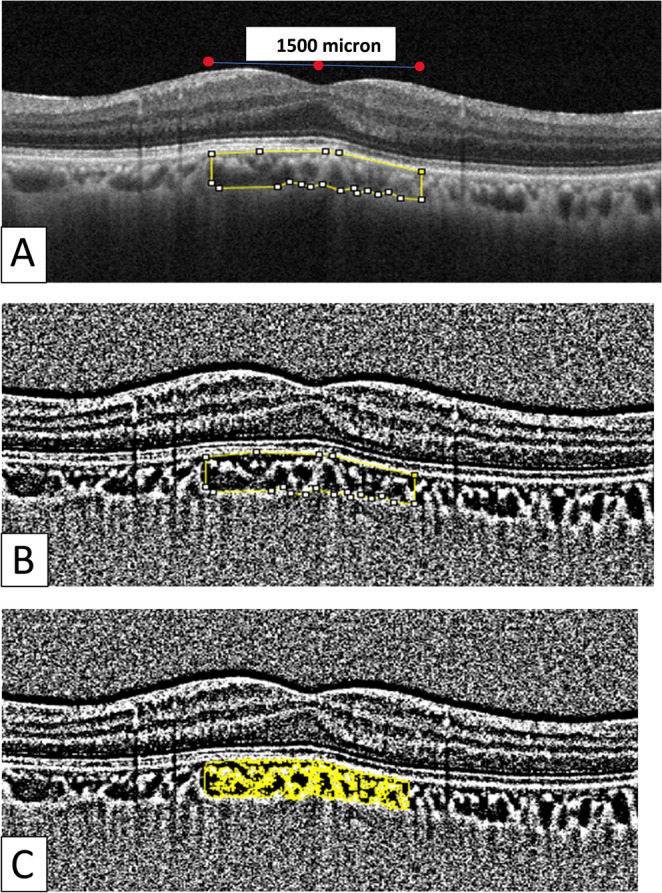
The subfoveal CVI was calculated using the ImageJ program. (**A**) An optical coherence tomography (OCT) scan of the subfoveal choroidal area beneath the fovea, with a width of 1500 microns, centered on the fovea. (**B**) The image in A was binarized using the Niblack automatic local threshold. (**C**) The vascular structures are identified using the color threshold and combined with the subfoveal area in the binarized image shown in B.

The subfoveal choroidal vascular index (SFCVI) values of the patients were measured using ImageJ, an image-processing and analysis program. The following link provides access to the ImageJ digital image analysis and processing software developed at the National Institutes of Health: https://imagej.nih.gov/ij/(13). The SD-OCT images were imported into ImageJ. The “Line” tool was selected, and the length scale of the SD-OCT image was set to the number of pixels. The images were converted into an 8-bit format. The Niblack ‘automatic local threshold’ method was utilized to facilitate a more straightforward selection of the choroid and scleral interface. The “polygon” tool was employed to delineate the “region of interest (ROI)” from the acquired OCT images. The outer border of the retinal pigment epithelial band and the border of the choroid-scleral junction were selected as the upper and lower borders of the choroid, respectively. For each image, a 1500-micron-wide subfoveal area was selected with the central fovea serving as the center point. The first bar in the color threshold was set to 0, and the second bar was set to 254 to enhance the vascular bed visibility. The second image was then saved in the ROI manager. These two images were then integrated, and the initial and final areas were measured using the ROI manager. The final area (subfoveal vascular area) was divided by the initial area (subfoveal total choroidal area) to obtain the SFCVI (Fig. 1).

### Statistical analysis

SPSS 28.0 was used for the analysis. The data were analyzed using descriptive statistics, including the mean, standard deviation, median, minimum, maximum, frequency, and ratio values. The normal distribution of variables was evaluated using the Shapiro-Wilk and Kolmogorov-Smirnov tests. The Mann-Whitney U test was used to analyze quantitative independent data with a non-normal distribution. The Wilcoxon test was used to analyze the dependent quantitative data. The Friedman exact test was used for repeated measurements. Qualitative independent data were analyzed using the chi-square test. Spearman’s correlation coefficient was employed to assess the relationship between the variables of interest. Statistical significance was set at *p* < 0.05.

## Results

The study included 28 patients who underwent PPV for RRD. The mean age of the patients was 61.21 ± 12.37 years, and 17 (60.7%) were male and 11 (39.3%) were female. A total of 28 eyes were operated on for RRD; 15 were on the right side (53.6%), while 13 were on the left side (46.4%). The mean interval between the onset of symptoms and surgical intervention was 5.11 ± 1.72 days. The prevalence of eyes with a solitary retinal tear was 17 (60.7%), whereas 12 (39.3%) exhibited more than one retinal tear. The most prevalent anatomical location of retinal tears was the superior temporal quadrant (39.3%), followed by the superior nasal (26.7%), inferior temporal (20%), and inferior nasal quadrants (6.6%). Macular involvement was observed in 27 (96.4%) patients with retinal detachment (RD).

In the case group, 1,000 cSt silicone oil was used as a tamponade agent in 16 cases (57.1%), whereas perfluoropropane gas (12–14% C_3_F_8_) was employed in 12 cases (42.9%). In patients in whom silicone oil was used as a tamponade, the mean interval from the initial surgical intervention to the subsequent removal of silicone oil was 56.88 ± 18.18 days (range, 23–80 days). The demographic and clinical characteristics of the patients are presented in Table 1.

**Table 1:** Demographic and clinical characteristics of the patients

		Silicone Oil (16 eyes)		C_3_F_8_ (12 eyes)		*p*
		Mean ± SD		Mean ± SD		
Age (Years)		61.75±14.16		60.50±10.05		0.797^m^
Axial Length (mm)		23.96±1.2		23.88±1.3		0.860^m^
Gender	Female	4	27.8%	7	58.3%	0.094^X2^
	Male	12	72.2%	5	41.7%	
Number of Retinal Tears	1	7	43.7%	10	83.3%	***0.016***^X2^
	2	5	31.2%	2	16.7%	
	3	3	18.7%	0	0.0%	
	4	1	6.2%	0	0.0%	

^m^Mann-whitney u test/^X2^Fisher’s exact test

No statistically significant differences were observed between the silicone oil and C_3_F_8_ tamponade groups with regard to age, sex, systemic comorbidities, side of the operated eye, and axial length discrepancy (*p* > 0.05). A statistically significant difference was observed in the number of retinal tears, with a lower number of cases observed in the C_3_F_8_ tamponade group compared to the silicone group (*p* = 0.016).

The preoperative BCVA was 1.39 ± 1.15 logMAR, 0.53 ± 0.31 logMAR at postoperative 1 month, 0.43 ± 0.39 logMAR at postoperative 3 months, and 0.32 ± 0.30 logMAR at postoperative 6 months in C_3_F_8_ group. The preoperative BCVA was 1.43 ± 0.99 logMAR, 0.61 ± 0.31 logMAR at postoperative 1 month, 0.44 ± 0.34 logMAR at postoperative 3 months, and 0.33 ± 0.33 logMAR at postoperative 6 months in silicone group. A significant enhancement in BCVA was observed at postoperative first, third, and sixth months compared to the preoperative period in eyes that underwent surgery for RRD (*p* < 0.05). Following the subdivision of the surgical eyes according to the utilization of silicone and C_3_F_8_ tamponade, no statistically significant differences were identified between the groups regarding BCVA at either the preoperative or postoperative control examinations (*p* > 0.05) (Table 2). Moreover, no statistically significant alterations in intraocular pressure (IOP) values were detected within silicone and C_3_F_8_ groups during the third- and sixth-months following surgery, compared to the initial IOP values recorded in the first month (*p* > 0.05). The mean intraocular pressure did not differ significantly between the silicone and gas groups at 1, 3, and 6 months (*p* = 0.241, 0.802, and 0.347, respectively).

**Table 2:** IOP, SFCT, SFCVI, and VA values in the silicone and C_3_F_8_ groups

		Case Group		*p*
		Silicone *n* = 16	C_3_F_8_*n* = 12	
IOP (mmHg)	1st month	16.44 ± 5.65	14.42±3.52	0.241^M^
	3rd month	13.75±4.13	14.25 ± 3.98	0.802^M^
	6th month	14.75 ± 5.27	15.92 ± 3.91	0.347^M^
p		0.247 ^F^	0.274 ^F^	
SFCT	1st month	225.44 ± 54.64*	232.58 ±42.19	*0.478^**M**^
	3rd month	231.50 ±50.99	231.50 ± 44.82	0.698^M^
	6th month	229.75 ± 55.69	224.17 ± 54.60	0.982^M^
p		0.984 ^F^	0.754 ^F^	
SFCVI	1st month	0.661±. 0.03*	0.630± 0.01	*0.015^**M**^
	3rd month	0.660± 0.03	0.637±02	**0.035**^**M**^
	6th month	0.662±0.02	0.649±0.03	0.205^M^
p		0.651 ^F^	0.205 ^F^	
VA (logMAR)	1st month	0.61±0.31	0.53±0.31	0.664^M^
	3rd month	0.44 ± 0.34	0.43±0.39	0.945^M^
	6th month	0.33±0.33	0.32±0.30	0.982^M^
		0.005^F^	0.005 ^F^	

*Measurements in the silicone subgroup at 1 month were excluded from analysis due to silicone-related measurement interference

^F^Friedman test for longitudinal subgroup comparison (1, 3, 6 months)

^M^Mann-whitney u test

Subfoveal choroidal thickness (SFCT) values were evaluated at the 1st month, 3rd month, and 6th month postoperatively. Measurements were taken in the first month in patients using silicone oil, but these were not included in the analysis, as silicone could affect the measurements. A comprehensive evaluation of the data revealed no statistically significant difference between the silicone and C_3_F_8_ groups with respect to postoperative third-, and sixth-month SFCT values (*p* = 0.698, and 0.982, respectively). In addition, SFCT showed no significant change in either the silicone group or the gas group in the 1st, 3rd, and 6th months (*p* = 0.984 in silicone group, and 0.754 in C_3_F_8_ group).

**Fig. 2:**
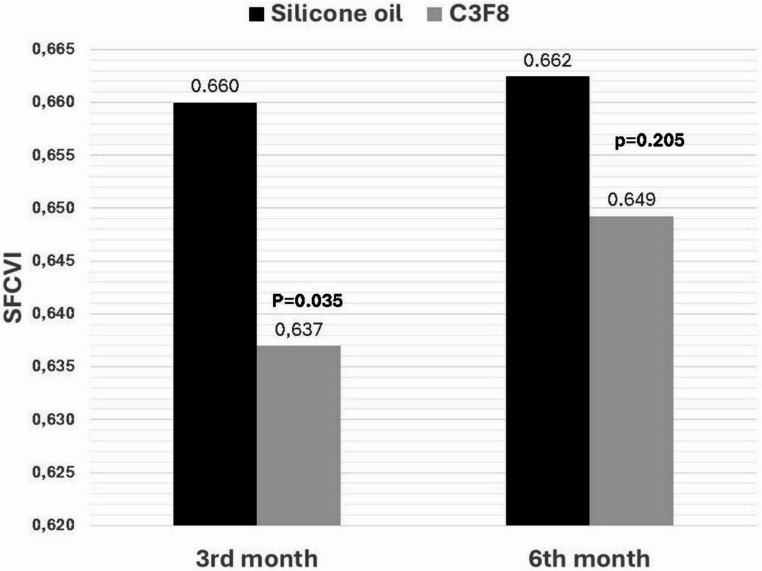
Histogram of SFCVI values in the silicone and gas groups

However, when comparing the monthly average SFCVI values in the silicone and gas groups, the values were lower in the gas group in the 1st and 3rd months, and a significant difference was observed between the groups (*p* = 0.015 and 0.035, respectively); however, this difference was not significant in the 6th month (*p* = 0.205). However, because silicon measurement could affect the measurements taken before silicon removal, we did not evaluate the difference in the 1st month (Table 2; Fig. 2).

When the changes in IOP, SFCVI, and SFCT values of the silicone and gas groups were measured both relative to the control eye and over months, SFCVI was significantly lower in the gas-treated eyes than in the control eyes of the same patients only in the first month (*p* = 0.003), while no significant differences were found in other periods and parameters (*p* > 0.05) (Table 3).

**Table 3:** IOP, SFCVI, and SFCT values measured monthly in the operated and healthy eyes of the silicone and gas groups and their statistical results

		Silicone oil *n* = 16			C_3_F_8_*n* = 12		
		Operated eye	Fellow eye	P*	Operated eye	Fellow eye	P*
IOP	1st month	16.44 ±5.65	13.13 ± 4.25	0.071	14.42 ± 3.52	15.08 ± 3.17	0.631
	3rd month	13.75 ± 4.13	14.25 ±3.45	0.713	14.25 ± 3.98	15.33 ± 3.42	0.482
	6th month	14.75 ± 5.27	14.31 ± 3.79	0.789	15.92 ± 3.91	15.67 ± 3.14	0.865
SFCVI	1st month	0.661 ± 0.038	0.666 ± 0.027	0.658	0.630 ± 0.013	0.656 ± 0.023	**0.003**
	3rd month	0.660 ± 0.030	0.667 ± 0.034	0.520	0.637 ± 0.022	0.656 ± 0.025	0.062
	6th month	0.662 ± 0.023	0.657 ± 0.022	0.506	0.649 ± 0.030	0.667 ± 0.042	0.246
SFCT	1st month	225.44 ±54.64	224.19 ± 43.58	0.943	232.58 ± 42.19	244.83 ± 32.00	0.432
	3rd month	231.50 ±50.99	235.63 ± 51.48	0.821	231.50 ± 44.82	239.33 ± 35.99	0.642
	6th month	229.75±55.69	234.88 ± 51.38	0.789	224.17 ± 54.60	233.42 ± 39.76	0.640

*The Wilcoxon signed-rank test

When the correlation between SFCVI and BCVA, IOP, and SFCT in the postoperative follow-up of eyes that underwent surgery due to RRD was evaluated, no significant correlation was observed between changes in SFCVI and other parameters (*p* > 0.05). When the correlation between the timing of silicone oil removal and postoperative IOP, SFCVI, SFCT, and VA values was analyzed, a statistically significant negative correlation was observed only between silicone oil removal time and SFCT at the sixth postoperative month (*r* =-0.629, *p* = 0.009). No significant correlations were found between the other parameters at the first, third, or sixth months (*p* > 0.05).

## Discussion

Rhegmatogenous retinal detachment is a condition in which the nutritional supply to the outer layers of the retina and photoreceptor layer is disrupted, resulting in the separation of the neurosensory retina from the RPE. This disruption leads to visual loss [15]. The choroid, a vital vascular structure situated beneath the RPE, plays a crucial role in supplying nutrients to the outer retinal layers [16]. Considering the important role of the choroid in supporting retinal homeostasis, there may be a correlation between the condition of the choroidal vasculature and visual acuity. EDI-OCT is a non-invasive method for cross-sectional imaging of the retina and choroid and is used to measure choroidal thickness with acceptable reproducibility and precision. In previous studies, choroidal thickness has been used as an indicator of the choroidal vascular structure. However, although choroidal thickness is a valuable measurement, it shows all choroidal vessels without discriminating between stromal and luminal vascular structures [17]. Moreover, choroidal thickness is affected by many factors, including axial length, age, myopia, and ethnicity. Enhanced visualization of the choroid has facilitated a more precise quantitative analysis of the choroidal vasculature. The CVI, first introduced by Agrawal et al. in 2016, is a relatively recent biomarker of the choroidal vasculature [13]. The development of CVI was predicated on the need for a more precise and robust evaluation of the choroidal vasculature. Recent studies have indicated that CVI may serve as a more effective biomarker for the diagnosis and monitoring of retinal diseases [17].

A substantial body of research in the extant literature has reported a decrease in choroidal thickness following retinal detachment surgery. In a study by Odrobina et al., choroidal thickness decreased at 3 and 6 months in patients diagnosed with proliferative retinal detachment who underwent treatment with 1000 cts silicone oil tamponade [6]. Karimi et al. reported a significant decrease in subfoveal choroidal thickness in eyes with long-term (i.e., ≥ 6 months) intravitreal silicone oil tamponade. This thinning did not improve after silicone oil removal [7]. Muslubaş et al. reported that patients diagnosed with new-onset RRD exhibited increased choroidal thickness prior to undergoing PPV. Following PPV, a reduction in choroidal thickness was observed [9]. Trivizki et al. reported a statistically significant decrease in choroidal thickness three months after surgery for retinal detachment in patients who underwent surgery. The study utilized C_3_F_8_ and 5500 cts silicone oil as tamponade agents [18]. In the present study, 1000 cts silicone and C_3_F_8_ gas were utilized as tamponade, and it was ascertained that choroidal thickness remained unchanged in both groups during the postoperative 6-month period. In addition, correlation analysis revealed a statistically significant negative correlation between the silicone oil removal time and SFCT at the sixth postoperative month. The observed variability in choroidal thickness in these studies may be attributable to variations in the density of the silicone oil used or the duration of its presence in the eye. To prevent the chronic effect of silicone oil on choroidal thickness, it seems appropriate to remove the silicone oil from the eye after it has been left in the eye for a sufficient period of time approximately 3 months.

Few studies has been conducted that have investigated changes in CVI in patients who underwent PPV for RRD and achieved anatomical success. Quiroz-Reyes et al. specifically examined the relationship between CVI values and the treatment modalities employed for eyes with RRD. They observed that eyes treated with vitrectomy alone exhibited higher CVI values, whereas those treated with a combination of scleral buckling (SB) and vitrectomy demonstrated lower CVI values [19]. It has also been reported that the correlation between CVI and BCVA indicates the important role that the choroidal vasculature plays in visual function [19]. Cho et al. found that preoperative SFCVI was statistically higher than postoperative 3-month SFCVI in the retinal detachment group without macular involvement (67.62 ± 2.35 vs. 65.84 ± 3.04, respectively; *p* = 0.009). Conversely, in the retinal detachment group with macular involvement, preoperative SFCVI exhibited a statistically significant decrease at 3 months postoperatively (64.01 ± 3.21 vs. 66.69 ± 2.64, respectively; *p* = 0.001) [20]. However, in this study, the authors performed not only PPV but also scleral buckling and combined surgeries.

In the present study, SFCVI values calculated in the postoperative control examinations of eyes that underwent successful PPV for RRD were compared with those of the unoperated fellow eyes. The SFCVI values decreased in the operated eyes compared to the control group at the first postoperative month in C_3_F_8_ group, but no significant difference was observed between the groups at the third and sixth months. The results of the present study are corroborated by the results of the study by Xia et al., in which 97 eyes of 97 patients diagnosed with idiopathic macular holes underwent vitrectomy with a 25 G needle and air tamponade [21]. A comprehensive analysis of the CVI alterations in patients before and after surgery was conducted. In addition, a comparative analysis was performed on the postoperative CVI changes in the patients and their fellow eyes. A significant decrease in the CVI value was found in the first postoperative week, and a gradual increase in the CVI value towards the initial levels over time was observed [21]. It was hypothesized that the alterations observed in CVI may be attributable to factors such as air tamponade, pressure fluctuations, and photoreceptor metabolism [21]. Sato et al. reported that ocular microcirculation was normal six months after scleral buckling or vitrectomy for RRD. However, the use of gas tamponade may have subclinical adverse effects on the circulation and neuroretinal disc rims [22].

In the present study, the SFCVI value in the C_3_F_8_ group at the first and third months was significantly lower than that of the silicone group (*p* = 0.015 and 0.035, respectively), but no significant difference was observed at the sixth month (*p* = 0.205).However, because silicone oil could affect the measurements, we did not evaluate the difference in the 1st month and take into consideration. In the C_3_F_8_ group, the SFCVI value at the first month after surgery was significantly lower than that in the control group. The study demonstrated that The C_3_F_8_ group exhibited a decline in SFCVI during the early postoperative period, followed by a gradual rise in SFCVI in subsequent examinations. This result may be explained by the high-pressure effect of C_3_F_8_ tamponade owing to its high interface tension (70 nm/m), the pressure of C_3_F_8_ on the retina, and choroidal blood flow owing to the prone position of the patients for a while after the operation. Decreased choroidal blood flow may cause a decrease in CVI. It was hypothesized that, given that the complete withdrawal time of C_3_F_8_ is approximately two months, the CFCVI would gradually increase after approximately two months. In the silicone group, SFCVI values at the first, third and sixth months after surgery did not differ significantly compared to the control group, and no significant change was observed in consecutive measurements. Chen et al. found that the CVI measured with silicone oil tamponade was lower in eyes undergoing RRD surgery [23]. In the aforementioned study, silicone oil tamponade decreased CVI. Furthermore, it was demonstrated that a longer tamponade period resulted in a greater decrease in CVI. In addition, choroidal blood circulation decreases in patients with retinal detachment with silicone oil tamponade [23]. However, in the aforementioned study, 5000cSt silicone oil was used and remained in the eye for approximately 3.5 months. In the present study, 1000cSt silicone oil was used as a silicone tamponade and remained in the eye for an average period of 2.5 months. The divergent outcomes observed across studies can be attributed to the utilization of disparate silicone oil tamponade agents and the varying retention times of silicone oil within the ocular cavity. The present study did not identify a significant correlation between silicone removal time and SFCVI.

A correlation was identified between the choroidal vasculature and visual outcomes in eyes with RRD in the postoperative period. The results demonstrated that a higher CVI was associated with better visual acuity, and CVI may be a potential biomarker for visual function [19]. Conversely, another study found that elevated CVI and silicone oil tamponade were associated with diminished postoperative BCVA [24]. In the present study, no significant correlation was found between the postoperative changes in SFCVI and postoperative BCVA in eyes that underwent RRD surgery.

The limitations of this study are as follows: first, the design was retrospective; second, the sample size was small and it was a single-center study; thirdly, because the sample size was small, we could not evaluate the correlation analyses separately for the gas and silicon groups; fourth, it was not possible to compare preoperative and postoperative changes in SFCVI because it was not possible to obtain preoperative OCT imaging of the macular region with adequate acquisition quality due to retinal detachment.

In conclusion, the subfoveal choroidal vascular index (SFCVI) shows a decrease in the early postoperative period followed by a return to normal levels in the late postoperative period in eyes that underwent pars plana vitrectomy for rhegmatogenous retinal detachment (RRD) and used C_3_F_8_ gas as tamponade. The SFCVI level may remain at a similar level post-surgery without significant change in eyes treated with silicone tamponade. Further research is needed to evaluate whether postoperative changes in SFCVI can be a prognostic factor for visual acuity improvement, the importance of the relationship between SFCT and SFCVI, and the relationship between different surgical techniques, tamponades, and SFCVI. This evaluation requires a larger sample size and longer follow-up periods.

## Data Availability

No datasets were generated or analysed during the current study.
